# Crossing boundaries in conservation physiology

**DOI:** 10.1093/conphys/coy015

**Published:** 2018-03-23

**Authors:** Sean Tomlinson, Jodie L Rummer, Kevin R Hultine, Steven J Cooke

**Affiliations:** 1 School of Molecular & Life Sciences, Curtin University, Kent Street, Bentley Western Australia 6102, Australia; 2 Department of Biodiversity Conservation and Attractions, Kings Park Science, Kattij Place, Kings Park, Western Australia 6005, Australia; 3 Australian Research Council (ARC) Centre of Excellence for Coral Reef Studies, James Cook University, 1 James Cook Dr, Douglas Queensland 4811, Australia; 4 Department of Research, Conservation and Collections, Desert Botanical Garden, 1201 N Galvin Pkwy, Phoenix, AZ 85008, USA; 5 Department of Biology and Institute of Environmental Science, Canadian Centre for Evidence-Based Conservation and Environmental Management, Carleton University, 1125 Colonel By Dr, Ottawa, Ontario, Canada K1S 5B6; 6 Department of Biology, Fish Ecology and Q2 Conservation Physiology Laboratory, Carleton University, 1125 Colonel By Dr, Ottawa, Ontario, Canada K1S 5B6

Ecophysiology investigates the mechanisms underpinning the interactions between an organism and its environment (Block and Vannier, 1994). The audience of, and contributors to, ‘Conservation Physiology’ are well aware as to how intriguingly complex these interactions can be; yet, we tend to take this one step further. We do not aim just to understand these complexities in some abstract sense. We want our research to be ‘useful and relevant’ to society. A critical aspect of ecophysiology has always been that it can be researched in anthropogenic or managed environments (Feder and Block, 1991; Block and Vannier, 1994), and there is a long heritage that enmeshes physiological measurements with conservation interests (Wikelski and Cooke, 2006; Cooke *et al.*, 2013). Indeed, it has been recognized for well over two decades that the full potential of conservation science depends on the integration of diverse expertise (Cooke *et al.*, 2013), and between research and practice (Cooke and O’Connor, 2010). Yet is conservation physiology really a useful science, and have we truly begun to establish the levels of cross-disciplinary collaboration required to make it so? Furthermore, do these broad collaborations involving complex research themes fit within the practical urgency of a conservation crisis?

By the time something draws the attention of conservation practitioners it’s already in a state of crisis management, usually requiring immediate action (du Toit, 2010). Very rarely can we observe conservation challenges emerging at a slow enough pace such that we can track the threats over time and effectively develop evidence-based conservation responses. In some ways, this has always been touted as the prime strength of conservation physiology (Block and Vannier, 1994; Cooke and O’Connor, 2010; Cooke *et al.*, 2013), because the discipline focuses on cause-and-effect relationships and the mechanisms that underpin ecological patterns. In practice, conservation physiology can rapidly draw empirical evidence to support conservation activities through targeted research programs that produce quantitative expectations of biological responses to environmental change. Generalizing these hypotheses is challenging, however, because it often involves taking measurements that are made in artificial, controlled environments and interpreting as if they were occurring in the complicated and often unpredictable real world (Tomlinson *et al.*, 2014) or taking useful and insightful measurements of very complex processes in the field (Tomlinson *et al.*, 2014). Complex questions require complex investigations and a broad and complex skill set (Dick *et al.*, 2016). Typically, this is accomplished by conducting cross-disciplinary research in collaborative teams. Here, we discuss three ways in which conservation physiology is meeting the challenge of integrating diverse expertise to maximize its practical value: by crossing taxonomic boundaries, incorporating statistical approaches from other fields, and ultimately developing cutting-edge technologies to make suitable measurements of organisms in the field.

## Taxonomic boundaries

Many of the physiological processes that inform conservation physiology, such as respiration, energetics, thermal tolerance and homoeostatic water balance, to name a few, are basal to all life. Yet increasingly, as biologists and physiologists, we have become specialized along taxonomic lines (*sensu*Block and Vannier, 1994; O’Brien, 2012). This has led to idiosyncrasies where the use of some techniques or equipment overlooks a broader applicability due to the narrowed expectations of a given field of study. Respirometry, for example, is a broadly applicable tool that measures gas exchange between an organism and the environment (either the atmosphere or surrounding water, if the organism is aquatic). Nevertheless, its use is often constrained to one or the other due to ‘blind spots’ within disciplines, even though it is equally applicable to the measurement of plant photosynthesis (Adhikari and White, 2014; Álvarez-Yépiz *et al.*, 2014) and calculation of animal metabolic rates (Winwood-Smith *et al.*, 2015; Rummer *et al.*, 2016). For example, in applying a standard flow-through respirometry approach that is widespread in animal physiology, Dalziell and Tomlinson (2017) showed that metabolic rates of plant seeds can be accurately measured, which broadly indicated seed viability, potentially providing a non-destructive tool to assess storage success. Metabolic rates were also highly interspecifically variable, however, suggesting huge research potential in this field and potential insights into demographic bottlenecks in conservation programs that depend on seed storage and recruitment from those stored seeds.

Another area where there is clear overlap and need for greater taxonomic integration is with nutritional physiology. The Anthropocene is recognized not just for its changes to global climate, but to land use and biotic interactions (Ellis *et al.*, 2010). These interactions form the basis of ecological energetic cascades through ecosystems (Tomlinson *et al.*, 2014) and it is increasingly recognized that nutrition is highly relevant to conservation (Birnie-Gauvin *et al.*, 2017). Unsurprisingly, with plants as the foundation of most food webs, aspects of food quality have dramatic influence on higher-level consumers, and changing climate has different effects on plants in aquatic versus terrestrial environments (Cotrufo *et al.*, 1998; O’Reilly *et al.*, 2003; Beardall *et al.*, 2009). Researchers cannot study energetics or food consumption in a grazer without thinking carefully about what, where, and when the animal of interest is grazing and how the changes in quantity and quality of forage reduces the quality of plant foods available to wildlife (Zvereva and Kozlov, 2006). Different grazing species also have different levels of resilience to such changes (Munn *et al.*, 2009; Munn *et al.*, 2012; Munn *et al.*, 2013), and introduced species can exacerbate ecological energetic effects of nutritional physiology (Birnie-Gauvin *et al.*, 2017). However, these cascades can be much more cryptic, imposing energetic barriers on passage through fragmented landscapes, which, when understood, provide empirical guidelines to their management and restoration (Tomlinson *et al.*, 2017a, 2017b). Critical to such studies is a balance between supply (i.e. the energy available to animals by plants) and demand (i.e. the requirements the animals have to persist and move through an environment), both of which change in response to changing environments (Tomlinson *et al.*, 2014; Birnie-Gauvin *et al.*, 2017) and require investigating both plant and animal ecophysiology.

## Statistical boundaries

When we are investigating cause-and-effect relationships between environmental change and biological processes, understanding, quantifying and parameterizing the physiological response of an organism is often critical (Carey, 2005; Cooke and O’Connor, 2010; Cooke *et al.*, 2013). Physiological responses tend to be non-linear, often asymmetrical, and generally unimodal (Angilletta, 2006; Flowers and Colmer, 2008), but ecophysiological studies have traditionally applied statistical transformations to create and visualize linear relationships for quantitative interrogation. In applying non-linear regression techniques and a series of functions that are well-established in ecotoxicology, Lewandrowski *et al.* (2016) have begun to quantify hydrothermal limits at critical life history stages of plant populations targeted for ecological restoration. These analyses provide a more subtle understanding of post-germination constraints to ecological restoration than are available from simple, linear response functions, offering more realistic empirical guidelines to conservation activity, including the potential for phenotypic flexibility in the face of chronic environmental changes.

One of the greatest challenges that we face as conservation physiologists is quantifying the effects of global climate change (Thomas *et al.*, 2004; Pörtner and Farrell, 2008). Efforts to understand how species will respond to climate change have often been pursued by ecologists using correlative approaches that are relatively independent from the mechanism that is potentially under selective pressure (Evans *et al.*, 2015). Moreover, it is already abundantly clear that there is an ‘art’ to modelling the ultimate distribution of range-shifting species in this way (Elith *et al.*, 2010). Congruent with Soberón and Nakamura’s (2009) conceptualization that responses to the abiotic environment are only one component that defines the realized niche, recent analyses have shown the powerful effects of changing biodiversity patterns and biotic interactions caused by climate change and the shortfalls that result in overlooking these (Pecl *et al.*, 2017). There are, however, emerging techniques that incorporate physiological processes into the complex statistical environment of biogeography and biophysics (Sutherst and Maywald, 1999; Kearney and Porter, 2016). Currently the two leading niche envelope models tend to be separated into animal-oriented (Kearney and Porter, 2016) and plant-oriented (Sutherst and Maywald, 1999) camps. However, the practical value of niche models for developing management policies is often data limited, restricted to a comparatively small number of well-studied organisms (Evans *et al.*, 2015), and substantial work is still required to evaluate model skill (Kish *et al.*, 2016). Furthermore, Kish *et al.* (2016) looked far afield and borrowed model testing approaches common in meteorology to understand the errors in their biophysical ecology models, and it has been suggested that similar liberalism may be required to parameterize mechanistic models for a broad array of taxa (Evans *et al.*, 2015).

Insofar as physiology influences ecology, so too does ecology influence physiology. While this may seem as intransigent as the question about the chicken and the egg, there is an emerging recognition that physiological constraints and influences can be and should be incorporated into a number of ecological modelling processes to better understand how physiological traits influence ecological patterns and conservation challenges (Bourbonnais *et al.*, 2014; Jachowski and Singh, 2015; Tarszisz *et al.*, 2018). Understanding home ranges and habitat use are critical to developing flexible and adaptable management programs at effective spatial scales for threatened species. Yet, the physiological components underpinning movement ecology and space use, ranging from the anthropogenic antagonism of stress (Bourbonnais *et al.*, 2014) to the ecological service of seed dispersal based upon digestive physiology (Tarszisz *et al.*, 2018), are only just now being explored. Interestingly, these concepts were raised decades ago by Huey (1991) in a paper titled ‘the physiological consequences of habitat selection’ but only today are we conducting the ecophysiology-grounded empirical research to understand relationships between physiology and ecological processes.

## Technological boundaries

The greatest constraint to measuring physiological processes, especially *in situ*, has always been limitations for measuring stress tolerance, energy fluxes and productivity at rapid temporal scales (Homyack, 2010). The measurements that we make are often complex, intricate and require fairly specific technologies to measure explicit indicators of physiological processes. Rarely do these approaches result in readily transportable technology that can easily provide measurements of organisms in their natural environment. Novel technological applications can, however, provide great insights. Approaches using radioisotopes have long been suggested a convenient means for estimating metabolic rates in very small animals. Indeed, a recent study used ^86^Rb turnover to determine that insect pollination in highly disturbed landscapes can be disrupted where the high cost of movement may not be offset by suitable food resources (Tomlinson *et al.*, 2017a). Measuring ecological energetics has a long history of informing management of species, but the same techniques are less reliable or ineffectual in relatively rare, secretive or wide-ranging marine predators. In response, Gallagher *et al.* (2017) applied a unique endocrinological approach to infer the energetic status of free ranging sharks, finding seasonal patterns in metabolic rates and nutritional balance that varied depending on the ecology of the species, but may imply management insights constructed around the seasonal patterns of stress and energetic challenge in marine apex predators. The tools that Gallagher *et al.* (2017) and Tomlinson *et al.* (2017a) developed have provided insight into possible ecological, biological and environmental factors contributing to the energetic bases of animal movement and possibly population densities, but have also raised many more research questions that will be required to refine these tools.

As well as energetic measurements, conservation biologists have used a broader range of physiological indices to understand habitat quality and threatening processes, but many such techniques no longer receive widespread application (Homyack, 2010). Biotelemetry has the advantage beyond simple measures of ecological energetics in that it links movement ecology with conservation physiology, providing a mechanistic element to the spatial evidence supporting conservation policy (Cooke *et al.*, 2004; Cooke, 2008). Most commonly, biotelemeters have been used to link field metabolic rates with movement patterns, and sometimes with feeding behaviours (Cooke *et al.*, 2004; Homyack, 2010). However, contributors to ‘Conservation Physiology’ have begun, in an array of taxa, much more subtle applications that are capable of assessing the impact of capture on physiological stress and survivorship of individuals and their movement patterns (French *et al.*, 2015; Gutowsky *et al.*, 2017). Critically, this approach takes existing technology and applies it so that physiological traits can be measured—sometimes for the first time—and results can be used to advise management of threatened species.

As a caveat, while novel technologies can be informative to physiological processes and provide mechanistic insights into conservation, there are also a number of emerging technologies that claim to offer amazing insights, but the value of which remain elusive. For example, CRISPR genome editing (Ran *et al.*, 2013), phenomics (Houle *et al.*, 2010) and metabarcoding (Thomsen and Willerslev, 2015) have all been suggested as ways in which we can identify or even modify critical physiological processes in complex ecosystems or in response to complex ecological challenges. Critically, if these advanced techniques prove to be reliable, they provide a vital link between the physiological traits that we understand to be adaptive now, and a capacity to ‘direct’ evolution in the face of conservation threats. It is precisely this link, however, that raises concerns: the adaptive nature of the physiology that we measure now, much in the way of the Spandrels and San Marco (Gould and Lewontin, 1979), does not necessarily represent the use for which it first evolved. If, as conservation physiologists, we want our research to result in successful conservation outcomes, we also need to be somewhat Hippocratic in our approach: before we apply the outcomes of our research, we must be certain that the technology that we employ works as we intended rather than creating new conservation problems (Nuñez *et al.*, 2017).

## Elegant simplicity

There is a consistent theme to most of the empirical studies that we have cited here; they take existing ideas, concepts, hypotheses, techniques, and technologies and apply them outside their usual scope. In doing so, the physiology crosses disciplinary boundaries between physiology and ecology, between botany and zoology, and between biology and the human dimension in ways that provide the evidence base around which adaptive management frameworks can be constructed (Cooke *et al.*, 2017; Nguyen *et al.*, 2017). The growing number of success stories in Conservation Physiology (Madliger *et al.*, 2017) have a number of common features, one of which is working across complex boundaries (disciplinary, taxonomic, scale, jurisdictions). This is the critical value to Conservation Physiology and the work published here; the insights gained have broad, practical value that crosses boundaries.
